# Transcriptomic Analysis of MGF360–4L Mediated Regulation in African Swine Fever Virus-Infected Porcine Alveolar Macrophages

**DOI:** 10.3390/ani15203029

**Published:** 2025-10-19

**Authors:** Zhen Wang, Liqi Zhu, Peng Zhao, Ying Huang, Chunhao Tao, Hong Jia

**Affiliations:** 1 Institute of Animal Sciences, Chinese Academy of Agricultural Sciences, Beijing 100193, China; wz2893963594@126.com (Z.W.); hy811cysbm@163.com (Y.H.); chunhao_tao@163.com (C.T.); 2 College of Veterinary Medicine, Shandong Agricultural University, Tai’an 271018, China; zhaopeng@sdau.edu.cn; 3 Institute of Comparative Medicine, College of Veterinary Medicine, Yangzhou University, Yangzhou 225009, China; 007119@yzu.edu.cn

**Keywords:** Aminoacyl-tRNA, ASFV, MGF360–4L, RNA-seq

## Abstract

African swine fever (ASF) is a highly contagious and virulent infectious disease caused by the African swine fever virus (ASFV), with mortality rates approaching 100% in domestic pigs. The global spread of ASF has caused enormous losses to the swine industry and species diversity, seriously affecting food safety in China. ASFV mainly infects the mononuclear system, inducing significant alterations in host-cell gene expression. Multigene family 360 (MGF360) genes play crucial roles in ASFV infection. To investigate the function of MGF360–4L, this study employed high-throughput RNA sequencing to analyze dynamic transcriptomic changes in porcine alveolar macrophages (PAMs) infected with wild-type ASFV (ASFV-WT) or MGF360–4L deletion mutant (ASFVΔMGF360–4L). Results demonstrated that both viruses activated host innate immune responses during early infection, significantly upregulating immune-related genes. At 16 h post-infection, differentially expressed genes in ASFV-WT- and ASFVΔMGF360–4L-infected cells were enriched in aminoacyl-tRNA biosynthesis pathways, suggesting a potential involvement of MGF360–4L in this process. This study elucidates novel ASFV–host interactions using transcriptomics, providing data to support ASF control strategies.

## Simple Summary

To investigate the function of MGF360–4L, this study performed transcriptomic analysis on porcine alveolar macrophages infected with ASFV-WT and ASFVΔMGF360–4L. The results showed that ASFV-WT and ASFVΔMGF360–4L activated host innate immune responses during early infection, significantly upregulating immune-related genes. At 16 h post-infection, differentially expressed genes in ASFV-WT- and ASFVΔMGF360–4L-infected cells were enriched in aminoacyl-tRNA biosynthesis, thus providing a certain reference for the prevention and control of ASFV.

## 1. Introduction

African swine fever (ASF), caused by African swine fever virus (ASFV), is a devastating hemorrhagic disease, affecting domestic pigs and wild suids with near-100% mortality in acute infections [1,2]. ASFV is a member of the Asfarviridae family within the nucleocytoviricote lineage [3]. ASFV exhibits a complex architecture: its virion comprises five concentric layers—a nucleoid core, core shell, inner envelope, icosahedral capsid, and host-derived outer envelope [4]. Critically, while both enveloped and non-enveloped virions remain infectious [5,6], enveloped particles exhibit significantly enhanced infectivity due to host membrane incorporation during budding. The ASFV genome consists of a linear, double-stranded DNA molecule (170–194 kb) with terminal hairpin loops formed by complementary 37-nucleotide palindromes [7]. This genome encodes 151–167 open reading frames (ORFs) organized into three distinct regions: (i) a central conserved segment (~125 kb) containing essential replication genes; (ii) highly variable terminal regions (left: 38–47 kb; right: 13–16 kb) enriched in multigene families (MGFs) and tandem repeats; and (iii) inverted terminal repeats (ITRs) flanking the genome ends [8,9,10,11]. Notably, the MGFs—particularly the MGF360 family—are implicated in host immune evasion, though the functional roles of individual members (e.g. MGF360–4L) remain poorly characterized. This knowledge gap is critical, as terminal region variability directly influences viral pathogenesis and host adaptation.

The multigene families (MGFs) of ASFV are closely related to the pathogenicity, host range, and tropism of ASFV. MGF360–4L is located in the left terminal repeat region of the ASFV genome and belongs to viral early proteins. Notably, during in vitro adaptation to continuous cell lines, genomic deletions frequently occur in MGF regions, including the deletion of MGF360 [12]. MGF360–4L harbors a receptor-interacting protein kinase homotypic interaction motif (RHIM) domain, which actively degrades RIPK3 in a dose-dependent manner to suppress necroptosis—a critical host defense pathway. Recent mechanistic studies showing that MGF360–4L degrades MDA5 via SQSTM1/p62-mediated autophagy and inhibits IRF3 phosphorylation; ASFV-ΔMGF-360–4L immunization provides protection against the lethal attack of ASFV-WT [13]. To define the transcriptional consequences of this immune subversion, we performed RNA-seq on porcine alveolar macrophages (PAMs)—the primary in vivo target of ASFV—infected with wild-type ASFV (ASFV-WT) or MGF360–4L-deleted virus (ASFVΔMGF360–4L) at 6 and 16 h post-infection (hpi). This dual-timepoint design captured early immune activation (6 hpi) and viral countermeasure deployment (16 hpi), addressing a critical gap in ASFV transcriptomics where prior studies predominantly focused on non-macrophage models. MGF360–4L-dependent reprogramming of host transcriptional networks was observed in this study, providing mechanistic insights into ASFV immune evasion strategies.

RNA-seq has emerged as the gold standard for dissecting virus–host interactions due to its unparalleled capacity to capture genome-wide transcriptional dynamics. While transcriptomic profiling has elucidated pathogenesis mechanisms for PRRSV [14], deltacoronavirus [15], and porcine pseudorabies virus [16], comprehensive time-resolved analyses of ASFV-infected primary macrophages—the virus’s in vivo cellular target—remain critically scarce. To address this gap, we performed RNA-seq on porcine alveolar macrophages (PAMs) infected with wild-type ASFV (ASFV-WT) or ΔMGF360–4L mutant at 6 and 16 h post-infection (hpi). This dual-timepoint strategy specifically captured (i) early host defense activation (6 hpi) and (ii) viral countermeasure deployment coinciding with MGF360–4L expression kinetics (16 hpi). This study intends to revel the mechanistic link between MGF360–4L and macrophage immune evasion, thereby providing references for the prevention and control of african swine fever virus.

## 2. Materials and Methods

### 2.1. Cells and Viruses

PAMs were isolated from 40- to 60-day-old specific-pathogen-free (SPF) pigs (Beijing SPF Swine Breeding and Management Center, China) via bronchoalveolar lavage with ice-cold PBS, as previously described [17]. PAMs were seeded in RPMI 1640 medium (Gibco) supplemented with 10% fetal bovine serum (FBS, Gibco, Thermo Fisher Scientific, Waltham, MA, USA) and 1% penicillin-streptomycin at 37 °C with 5% CO_2_. The recombinant ASFVΔMGF360–4L was created with deletion of the MGF360–4L gene from the ASFV JS strain (ASFV-WT) through homologous recombination, which can stabilize mCherry expression to facilitate infection monitoring. Genomic deletion was confirmed by Sanger sequencing. Both viruses were propagated in PAMs, aliquoted, and stored at − 80 °C in viral preservation medium (20% FBS, 10 mM HEPES in DMEM). All viral work was performed in the Animal Biosafety Level 3 (ABSL−3) facility at Yangzhou University (certification #CN-ABSL3–2021-YZU). Virus handling protocols were approved by the Yangzhou University Institutional Biosafety Committee (IBC Protocol #YU-IBS−2023-ASFV−07).

### 2.2. Preparation of Transcriptome Samples

PAMs were seeded in 10^6^ cells/well into 6-well plates overnight; PAMs were subsequently infected with ASFV at a multiplicity of infection (MOI) of 1. The study comprised three experimental groups: a mock group (uninfected control), AW group (wild-type ASFV-infected), and AC group (ASFVΔMGF360–4L-infected). All infections were performed in triplicate with independent biological replicates (*n* = 3) to ensure statistical power. The cells were collected at 6 h and 16 h post-infection and were lysed directly in wells with Trizol (Thermo Fisher Scientific, Waltham, MA, USA) and processed for RNA sequencing by Tsingke Biotechnology Co., Ltd. (Beijing, China).

### 2.3. Extraction and Quantification of Total RNA

Total RNA was extracted from Trizol^®^-lysed samples using the chloroform-isopropanol protocol as per the manufacturer’s instructions [18] (Thermo Fisher Scientific, Waltham, MA, USA), with on-column DNase I treatment (Qiagen, Cat# 79254) to eliminate genomic DNA contamination. RNA concentration and purity were quantified using a NanoDrop 2000 spectrophotometer (Thermo Fisher Scientific, Waltham, MA, USA). RNA integrity was rigorously assessed using an Agilent 2100 Bioanalyzer (Agilent Technologies, Santa Clara, CA, USA) with RNA 6000 Nano Chips, and samples with an RNA Integrity Number (RIN) ≥ 8.5 were retained for sequencing. Genomic DNA contamination was further validated by qPCR targeting porcine β-actin intron, amplification efficiency >95%, no-template controls negative. Only samples passing all QC thresholds were processed for library construction.

### 2.4. Construction of cDNA Library

The process of library construction was as follows:

1. Purification and fragmentation of mRNA.
2. Synthesis of the first strand of cDNA.
3. Synthesis of the second strand of cDNA and purification of the second strand products.
4. Perform end repair and 3′-end A-tailing on the above products, followed immediately by adapter ligation reaction.
5. Purification of ligation products and fragment selection.
6. PCR amplification and purification; Qsep−400 was used for quality inspection.

### 2.5. RNA Sequencing

Qualified samples were sequenced. The raw image data files obtained from high-throughput sequencing were analyzed by CASAVA base calling, which were then converted into raw sequencing data (raw data) and stored in FASTQ file format. This contained the sequence information of the sequencing data and the corresponding sequencing quality information. First, sequencing error rate distribution was analyzed; a higher base quality value indicates more reliable base recognition and greater accuracy. Sequencing base content distribution was then assessed. Raw data were processed for quality control using Trimmomatic software (version 0.36), and the resulting clean reads were used for subsequent analysis.

### 2.6. Sequence Alignment

The specified genome was used as the reference sequence for sequence alignment and analysis. The version of the reference genome used is Hisat2 (version 2.1.0) was used to align clean reads with the reference genome to obtain their positional information on the reference genes and unique sequence characterization.

### 2.7. Gene Expression Level Analysis

Transcript abundance directly reflects gene expression levels, with higher transcript abundance indicating greater gene expression. FPKM is a standardized index for measuring the expression level of transcripts or genes, and its calculation formula is as follows:

$$FPKM=\frac{cDNA\;Fragments}{Mapped\;Fragments\;\left(Millions\right)\times \;Transcript\;Length\;(kb)}$$

The gene expression levels calculated by the FPKM method can be directly used to compare differences in gene expression between different samples.

### 2.8. Differential Gene Expression Analysis

Differentially expressed genes (DEGs) between ASFV-WT- and ASFVΔMGF360–4L-infected PAMs were identified using DESeq2 v1.38.0 [19]. Pearson correlation coefficient (R) for biological replicates was used to evaluate the correction of biological replicates, the R^2^ value closer to 1 indicated stronger correlation between replicate samples. We set project thresholds to screen for differential genes; the screening criteria were fold change ≥ 1.5 and *p*-value < 0.05.

### 2.9. Bioinformatics Analysis

Gene Ontology (GO) is an internationally standardized gene function classification system that provides a dynamically updated controlled vocabulary to comprehensively describe the attributes of genes and their products in organisms [20]. The GO system categorizes gene functions into three primary domains: Molecular Function (MF), Cellular Component (CC), and Biological Process (BP). The Kyoto Encyclopedia of Genes and Genomes (KEGG) was mainly used to analyze the enriched pathways and related functions of DEGs [21].

## 3. Results

### 3.1. RNA Quality Detection

**Figure 1:**
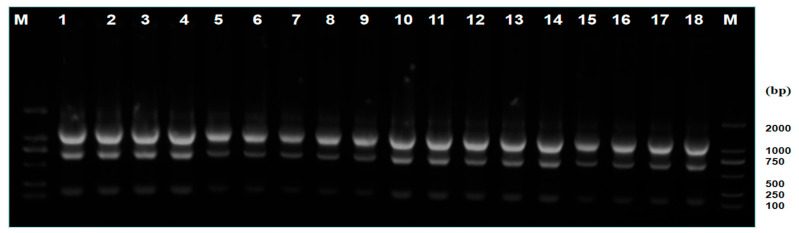
Quality assessment of RNA from ASFV-WT- and ASFVΔMGF360–4L-infected PAMs. M: DL2000 DNA Maker; lanes 1–3: mock 6 group 1–3; lanes 4–6: mock 16 group 1–3; lanes 7–9: AW 6 group 1–3; lanes 10–12: AW 16 group 1–3; lanes 13–15: AC 6 group 1–3; lanes 16–18: AC 16 group 1–3.

Quality control confirmed that the RNA integrity and concentration of all samples (Figure 1 and Table 1) were sufficient to construct two independent libraries. All samples reached grade A, meeting the quality requirements for cDNA library construction and sequencing, and could be normally used for cDNA library construction.

### 3.2. RNA-seq Data Quality Metrics

Quality assessment results are shown in Table 2. The average Q30 ratio across samples was over 95.45%, exceeding the minimum threshold of 85% required by the sequencing platform. Clean reads were aligned to the reference genome using Hisat2, with 92.49–96.12% of reads mapping successfully. Of these, 89.56–93.35% mapped to unique genomic positions, meeting subsequent analysis requirements.

### 3.3. Differential Expression Analysis of PAMs Infected with ASFV-WT and ASFVΔMGF360–4L

**Figure 2:**
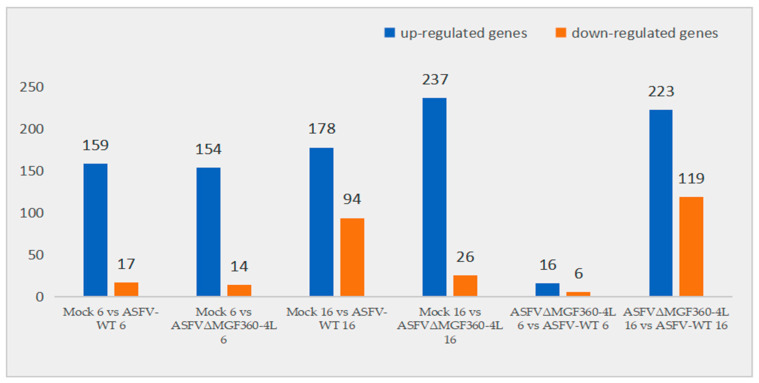
Statistics of the number of differentially expressed genes. The abscissa represents the name of each group of samples, and the ordinate represents the number of up-regulated and down-regulated genes.

The reads’ count values and expression levels of mRNA in each group of samples were obtained using DESeq2 software. The threshold criteria for screening differential genes were fold change ≥ 1.5 and *p*-value < 0.05. Comparative transcriptomic analysis revealed significant gene expression changes upon ASFV infection. At 6 hpi, the AW group exhibited 176 differentially expressed genes (DEGs) compared to the mock group (159 up-regulated and 17 down-regulated). Similarly, the AC group showed 168 DEGs (154 up-regulated and 14 down-regulated). At 16 hpi, there were 272 DEGs in the AW group (178 up-regulated genes and 94 down-regulated genes) and 263 DEGs in the AC group (237 up-regulated and 26 down-regulated), compared with the mock group. A comparison of the AW group with the AC group revealed the presence of 22 DEGs (16 up-regulated and 6 down-regulated) at 6 hpi, as well as 342 DEGs (223 up-regulated and 119 down-regulated) at 16 hpi, respectively (Figure 2).

**Figure 3:**
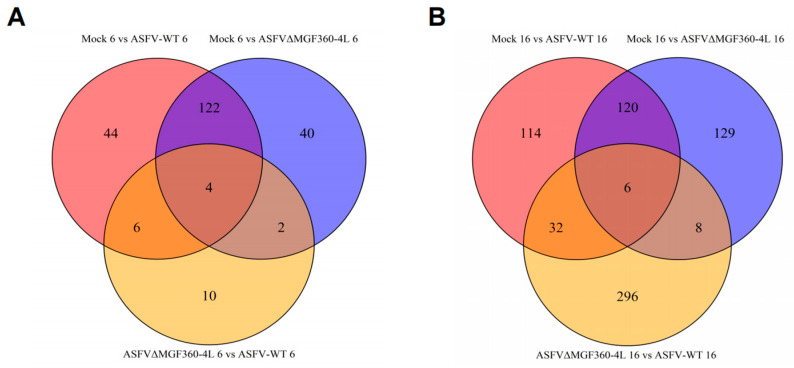
Shared and unique differentially expressed genes. (**A**) Shared and unique differentially expressed genes in the 6 h treatment group; (**B**) shared and unique differentially expressed genes in the 16 h treatment group.

The number of common and unique DEGs among comparison groups is shown in Figure 3. At 6 hpi, unique DEGs in the mock vs. AW groups included FOXF1, SKAP1, and FBXW7, whereas unique DEGs in the mock vs. AC groups included MAP7, BCL2L14, and FZDB. Common DEGs between these groups included the innate immunity-related genes IFIT1, IRF7, TLR3, and IL7R. Unique DEGs in the AC vs. AW groups included LRRC3, TIMP4, and UPF3A (Figure 3A). At 16 hpi, unique DEGs in the mock vs. AW groups included USP15, NR6A1, and ATAD1; unique DEGs in the mock vs. AC groups included ISG12, RRBP1, and TRIM25; common DEGs included IFI44L, ISG20, IRF7, and DTX3; and unique DEGs in the AC vs. AW groups included ZFHX2, HSPG2, and USP39 (Figure 3B).

### 3.4. GO Enrichment Analysis of Differentially Expressed Genes

The GO database was used to analyze the expression levels of DEGs after infection with AW and AC. The results showed that, compared with the mock group, the cellular components of the AW and AC group mainly included organelles, membranes, and membrane parts, etc.; the molecular functions involved mainly included binding, molecular sensor activity, and catalytic activity; and the biological processes involved mainly included cellular processes, single-organism processes, metabolic processes, immune system processes, etc.

### 3.5. KEGG Analysis of Differentially Expressed Genes

**Figure 4:**
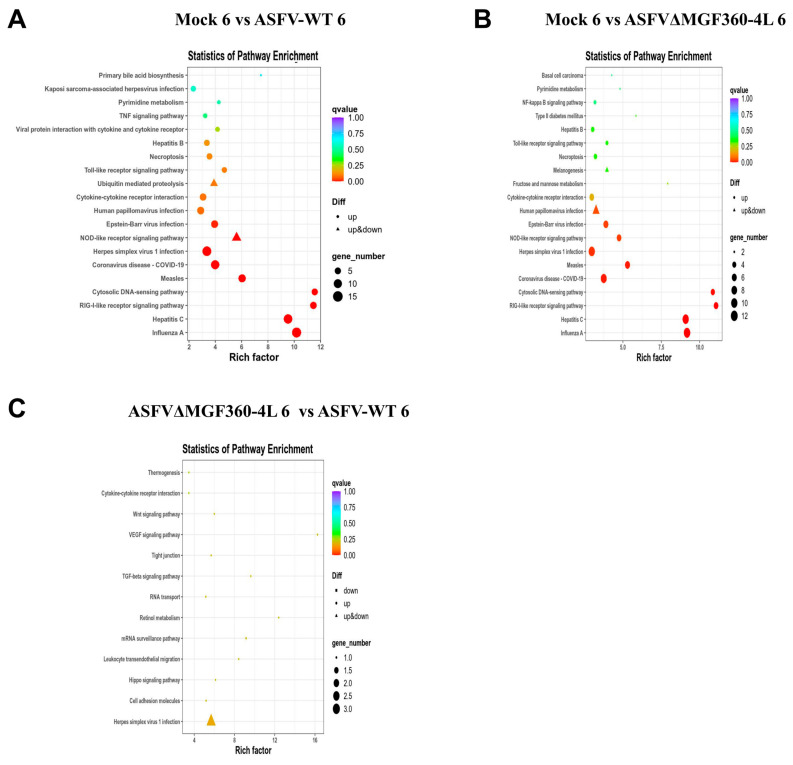
Scatter plot of KEGG enrichment pathways of differentially expressed genes in PAMs at 6 h post-infection. (**A**–**C**) The abscissa represents the enrichment factor, the ordinate represents the name of the enriched pathway, and the color indicates the *q*value. (**A**) mock 6 vs. ASFV-WT 6; (**B**) mock 6 vs. ASFVΔMGF360–4L 6; (**C**) ASFVΔMGF360–4L 6 vs. ASFV-WT 6.

At 6 hpi, the DEGs in both the AW and AC groups (vs. mock) were significantly enriched in immune-related pathways including RIG-I-like receptor, NOD-like receptor, toll-like receptor, TNF, and NF-κB signaling pathways. These DEGs were also enriched in disease-related pathways: influenza A, hepatitis C, herpes simplex virus 1 infection, and coronavirus disease (COVID−19) (Figure 4A,B). However, few DEGs existed between the AW and AC groups. In the AC vs. AW comparison at 6 hpi, DEGs showed significant enrichment in herpes simplex virus 1 infection, Hippo signaling, leukocyte transendothelial migration, TGF-β signaling pathway, and cell adhesion molecules (Figure 4C).

**Figure 5:**
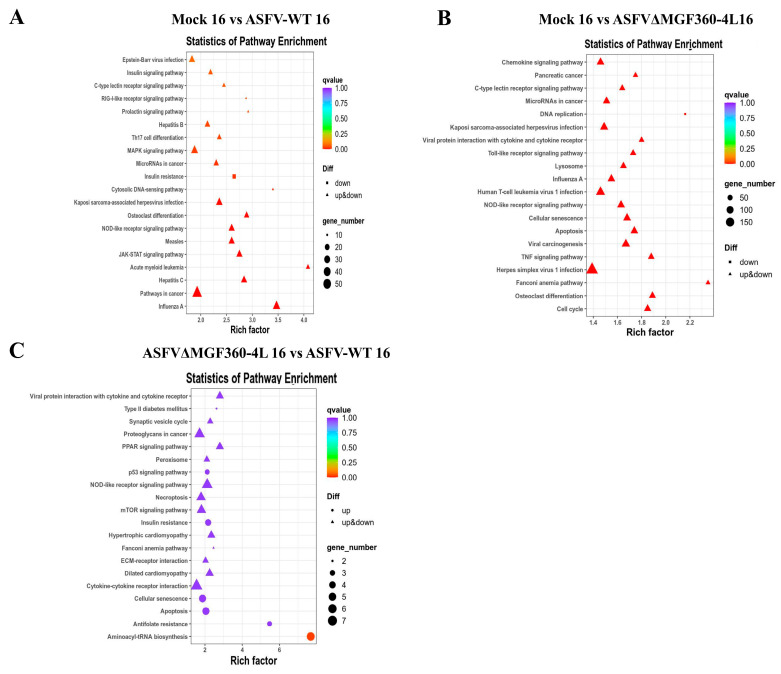
Scatter plot of KEGG enrichment pathways of differentially expressed genes in PAMs at 16 h post-infection. (**A**–**C**) The abscissa represents the enrichment factor, the ordinate represents the name of the enriched pathway, and the color indicates the *q*value. (**A**) mock 16 vs. ASFV-WT 16. (**B**) mock 16 vs. ASFVΔMGF360–4L 16. (**C**) ASFVΔMGF360–4L 16 vs. ASFV-WT 16.

At 16 hpi, DEGs in the AW group (vs. mock) showed significant enrichment in immune-related pathways: the JAK/STAT, NOD-like receptor, cytosolic DNA-sensing, MAPK, and RIG-I-like receptor signaling pathways. Enriched disease-related pathways included influenza A, hepatitis C, Kaposi’s sarcoma-associated herpesvirus infection, and Epstein–Barr virus infection (Figure 5A). DEGs in the AC group (vs. mock) were enriched in immune pathways, including the TNF, NOD-like receptor, toll-like receptor, C-type lectin receptor signaling pathways, and viral protein interactions with cytokine and cytokine receptors (Figure 5B). In the AC vs. AW comparison, DEGs were enriched in aminoacyl-tRNA biosynthesis, apoptosis, cellular senescence, cytokine-cytokine receptor interaction, extracellular matrix-receptor interaction, mTOR signaling, and P53 signaling pathways (Figure 5C).

These pathways play crucial roles in ASFV infection. At 6 hpi, few DEGs existed between the ASFV-WT- and ASFVΔMGF360–4L-infected groups. However, at 16 hpi, significant DEGs enrichment occurred in aminoacyl-tRNA biosynthesis.

### 3.6. Differential Expression of Host Innate Immune Genes in ASFV-Infected PAM Cells

**Figure 6:**
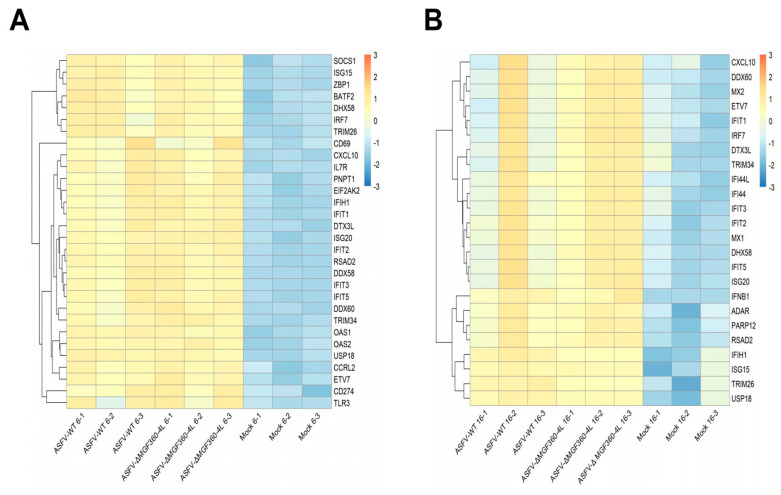
Cluster analysis of differential gene expression. (**A**) Cluster analysis of immune-related differential gene expression at 6 h post-infection. (**B**) Cluster analysis of immune-related differential gene expression at 16 h post-infection.

To further investigate the transcriptional profiles of immune-related genes in ASFV-infected PAMs, the heatmaps of immune-related genes were generated, aiming to reveal the differences between the AW and AC groups in inducing genes related to innate immune responses. The results showed that both AW and AC induce significant up-regulation of immune-related genes. At 6 hpi, the transcriptional level of the pattern recognition receptor TLR3 increased significantly. Upon ASFV infection, TLR3 recognizes the virus and trigger a series of immune responses. Meanwhile, the transcriptional levels of interferon-stimulated genes (ISGs) such as ISG15, ISG20, OAS1, OAS2, MX1, and MX2 were significantly up-regulated, and these ISGs play a crucial role in resisting viral infection. Additionally, the transcriptional levels of interferon-induced tetratricopeptide repeat family proteins (IFITs), including IFIT2, IFIT3, and IFIT5, were found to be significantly increased (Figure 6A). At 16 hpi, in addition to the increased transcriptional levels of ISGs, the mRNA level of IFNβ1 also significantly increased (Figure 6B). The above results demonstrated that after ASFV invades cells, it is recognized by the immune system, triggering an antiviral response.

## 4. Discussion

ASFV primarily infects the mononuclear system, which includes porcine bone marrow-derived macrophages and primary porcine alveolar macrophages. Following infection, ASFV alters host-cell gene expression patterns and regulates cellular processes through multiple mechanisms, disrupting normal physiological functions. Transcriptomic changes post-infection involve multifaceted responses that collectively establish a complex virus–host interaction network. In this study, PAMs were infected with ASFV-WT or ASFVΔMGF360–4L, with samples collected at 6 and 16 hpi for transcriptomic analysis. This approach aimed to reveal infection-induced transcriptomic changes and regulatory mechanisms. Bioinformatics analysis compares ASFV-infected groups with mock-infected controls to characterize early host–cell interactions.

Recent studies have shown that during the early stages of ASFV infection, host organs initiate a coordinated immune response by activating immune-related genes, triggering a proinflammatory cytokine storm, and inducing the activation of interferon pathways [22]; this phenomenon was also observed in this study. At 6 hpi, IFN-stimulated genes were significantly up-regulated. Interferons play an important role in antiviral defense by targeting multiple stages of viral replication. Previous studies demonstrated that OAS1 interacts with the major structural protein P72 of ASFV, recruiting TRIM21 through the ubiquitin-proteasome pathway to degrade P72 via K63-linked ubiquitination. This disrupts assembly of mature virions and inhibits ASFV replication [23]. In this study, OAS1 transcription was up-regulated at 6 and 16 hpi during ASFV infection compared to mock infection, indicating multifaceted host antiviral responses. The interferon-induced tetratricopeptide repeat (IFIT) protein family possesses broad-spectrum antiviral activity by inhibiting viral transcription and replication. Previous studies have shown that IFIT proteins interact with molecules such as MAVS, STING, and TBK1 to enhance IFN expression [24]. Following infection of PAMs with ASFV-WT and ASFVΔMGF360–4L, IFIT2, IFIT3, and IFIT5 transcription were significantly up-regulated. Further studies are required to determine whether IFIT proteins act as anti-ASFV effectors. In ASFV-WT- and ASFVΔMGF360–4L-infected cells. USP18 mRNA levels significantly increased at both 6 and 16 hpi. USP18 functions not only as a deubiquitinating enzyme but also as an effective inhibitor of IFN signaling, independent of its enzymatic activity. For example, classical swine fever virus (CSFV) up-regulates USP18 to inhibit the IFN-I pathway, evading innate immunity and promoting replication. ASFV similarly inhibits type I IFN signaling. Whether ASFV exploits USP18 as an immune evasion strategy merits systematic investigation. At 16 hpi, DEGs in ASFVΔMGF360–4L-infected cells (vs. parental strain) showed enrichment for aminoacyl-tRNA synthetases (AARS). AARS ensure translation fidelity by catalyzing tRNA aminoacylation and play essential roles in cellular processes. They also participate in RNA splicing, transcriptional regulation, translation, and homeostasis. Recent research provided novel evidence that the enzyme tryptophanyl-tRNA synthetase 2 (WASR2) facilitates PRV infection through its management of protein and lipid levels [25]. Whether aminoacyl-tRNA synthetase affects the replication and proliferation of ASFV still requires further investigation. The putative association inferred from enrichment analysis and the interaction between ASFV MGF360–4L and the host AARS warrant further investigation to elucidate molecular mechanisms.

Interactions between viruses and host cells are extremely complex. During early ASFV infection, the virus activates host innate immunity, significantly upregulating immune-related gene expression. This indicates that host cells adopt a series of measures to resist viral infections. At 6 hpi, deletion of MGF360–4L exerted a minimal impact on macrophage transcriptomes. However, at 16 hpi, MGF360–4L deficiency altered aminoacyl-tRNA biosynthesis-related expression. In summary, this study elucidated mRNA changes in ASFV-infected PAMs using RNA-seq, providing data to understand ASFV–host-cell interactions.

## 5. Conclusions

This study conducted transcriptomic analysis of ASFV-WT and ASFVΔMGF360-4L in PAMs. The results showed that both ASFV-WT and ASFVΔMGF360–4L could induce innate immune responses in host cells, thereby causing a significant up-regulation of immune-related genes. At 6 hpi, there were relatively few differently expressed genes between ASFV-WT-infected and ASFVΔMGF360-4L-infected groups; however, at 16 hpi, the differentially expressed gens between the two groups were significantly enriched in the aminoacyl-tRNA synthetase pathway. In the future, we will conduct research on the interaction between aminoacyl-tRNA synthetase and ASFV to clarify their specific mechanism of action.

## Figures and Tables

**Table 1:** The results of sample detection.

Sample Name	RNA Concentration (ng/µL)	RIN Value	Sample Grade
Mock6–1	207.3	9.74	A
Mock6–2	212.2	9.52	A
Mock6–3	233.3	9.72	A
AW6–1	178.6	9.71	A
AW6–2	242.9	9.70	A
AW6–3	202.8	9.71	A
AC6–1	170.2	9.38	A
AC6–2	208.0	9.69	A
AC6–3	220.8	9.68	A
Mock16–1	182.4	9.53	A
Mock16–2	209.7	9.72	A
Mock16–3	189.0	9.72	A
AW16–1	178.7	9.68	A
AW16–2	203.8	9.69	A
AW16–3	183.9	9.28	A
AC16–1	171.2	9.69	A
AC16–2	208.1	9.16	A
AC16–3	178.0	9.03	A

**Table 2:** Overview of RNA-seq results.

Sample Name	≥Q30 (%)	GC Content (%)	Clean Reads	Mapped Reads (%)	Unique Mapped Reads (%)
Mock6–1	96.25	50.48	33,044,634	96.12	93.34
Mock6–2	96.28	50.65	29,106,984	96.07	93.35
Mock6–3	96.09	50.82	33,290,771	95.88	93.09
Mock16–1	96.07	51.05	31,606,949	95.92	93.13
Mock16–2	96.11	51.20	28,476,041	95.66	92.71
Mock16–3	96.21	50.64	29,694,705	96.12	93.32
AW6–1	96.23	51.03	31,892,470	94.55	91.67
AW6–2	96.07	51.13	29,008,431	94.15	91.30
AW6–3	96.42	49.89	24,006,120	94.47	91.76
AW16–1	96.11	50.05	24,403,020	95.05	92.31
AW16–2	95.95	50.85	25,739,922	92.49	89.56
AW16–3	96.45	50.13	28,090,758	94.77	91.98
AC6–1	96.68	50.65	30,000,168	95.10	92.25
AC6–2	96.21	50.80	26,211,255	94.25	91.53
AC6–3	96.25	50.49	33,012,588	94.83	92.04
AC16–1	96.93	50.87	38,409,071	94.52	91.45
AC16–2	96.54	51.39	30,568,378	94.14	91.20
AC16–3	96.32	51.16	31,033,817	93.78	90.82

## Data Availability

All data generated for this study are included in the article.

## Abbreviations

ASF	African swine fever
ASFV	African swine fever virus
RNA	Ribonucleic acid
ORF	Open Reading Frame
RHIM	Receptor-interacting protein kinase homotypic interaction motif
SPF	Specific-pathogen-free
PAM	Porcine alveolar macrophages
DEG	Differential expression analysis
TNF	Tumor necrosis factor
CSFV	Classical swine fever virus
